# A new method for second toe flap revascularization in plantar dominant artery pattern - dorsal metatarsal artery interpositional graft

**DOI:** 10.55730/1300-0144.5562

**Published:** 2022-10-18

**Authors:** Erhan SÖNMEZ, Ersin AKŞAM, İlker UYAR

**Affiliations:** Department of Plastic, Reconstructive and Aesthetic Surgery, Faculty of Medicine, İzmir Kâtip Çelebi University, İzmir, Turkey

**Keywords:** Arterial graft, dominant arterial system, dorsalis pedis, toe to hand transfer

## Abstract

**Background/aim:**

Toe transfer to hand is a viable option for patients, which can provide functionally useful digits. Harvesting of the second toe is still accepted as a demanding surgical procedure. The major questions before this transfer are the location and the dominance of the arterial anatomy of the second toe.

The authors present the incidence of patients with a dominant plantar artery system and a description of a versatile technique that can be used for toe transfers in these patients.

**Materials and methods:**

The study was designed retrospectively. Toe to hand transfers performed between 2010 and 2018 were reviewed. The patients with a dominant plantar arterial system were included in this study. The dorsalis pedis arterial graft technique described by the authors was used in all cases with a dominant plantar system. All of transfers were done by the senior author. All cases followed up at least one year post-operatively. The survival of the transferred finger was examined in the follow-ups.

**Results:**

Eleven toe to hand transplantation cases in nine traumatic patients were included in this series. The reason for the operation was trauma in all patients. Second toe transfer was performed in all of the cases. Plantar dominant arterial system was seen in 3 of the 9 patients. Dorsal arterial system was dominant in the remaining six patients. Dorsalis pedis arterial graft technique was used in these four cases. All of the toes survived, and reexploration was needed in 2 cases because of venous insufficiency. Mean follow-up time was 16.4 months.

**Conclusion:**

This is the first study to recommend the use of dorsalis pedis as an arterial graft instead of vein grafts. This surgical method described will make these rarely performed transfers easier and affect the success rates positively.

## 1. Introduction

Traumatic loss or congenital absence of one or multiple digits of the hand is very disabling to an individual. Toe transfer to hand is a viable option for these patients, which can provide functionally useful digits and may enhance patient well-being without a major disability of the donor foot. Transfer of the first, second or combined second and third toes have been described, and among these, second toe transfer results in significantly less morbidity in the donor site from aesthetic and functional perspectives [1–3].

Harvesting of the second toe is still accepted as a demanding surgical procedure. The major questions before this transfer are the location and the dominance of the arterial anatomy of the second toe. The first dorsal metatarsal artery is capable of supplying the arterial circulation of the second toe in the majority (approximately 70%) of the cases, and the relationship between this artery with the interosseous muscle and the intermetatarsal ligament is of paramount importance in the flap dissection. It is widely accepted that it is much easier to harvest the second toe via solely dorsal approach without any plantar dissection, which is possible when the first dorsal metatarsal artery is capable of supplying the arterial circulation of the second toe [4].

Dominant plantar arterial system is found in about 30% of the toe transfer cases, and plantar arteries of the toes have to be used for reestablishing the arterial circulation of these toes [5]. Plantar dissection of the digital arteries of the toes is technically more challenging compared to the dorsal dissection. Although with appropriate retraction, plantar arterial system can be traced proximally to the metatarsal cleft, extensive plantar foot dissection can be associated with increased morbidity of the donor foot. Using vein graft is advised in order to eliminate this challenging dissection and prevent increased morbidity of the donor foot [5].

The authors present the incidence of patients with a dominant plantar artery system and a description of a versatile technique that can be used for toe transfers in these patients.

## 2. Materials and methods

The study was designed retrospectively. Local ethics committee approval was obtained for the study. The patients who underwent toe-to-hand finger transfer at the university hospital between 2010 and 2018 were reviewed. The patients with a dominant plantar arterial system were included in this study. Patients who did not attend the controls regularly and did not comply with the postoperative recommendations were excluded from the study. The dorsalis pedis arterial graft technique described by the authors was used in all cases with a dominant plantar system. All of transfers were done by the senior author (E.S). All cases followed up at least one year postoperatively. The survival of the transferred finger was examined in the follow-ups.

### 2.1. Surgical technique

The second toe dissection is initiated from the dorsal aspect of the first intermetatarsal space, identifying the medial digital artery of the second toe which arises from the first metatarsal artery. The dominance of the dorsal or plantar arterial system can be determined at this time. The dissection is straightforward when the dorsal system is dominant. Dorso-plantar connections are ligated, and the proximal dissection of the dorsalis pedis artery is continued until required pedicle length is gained [5].

**Figure 1:**
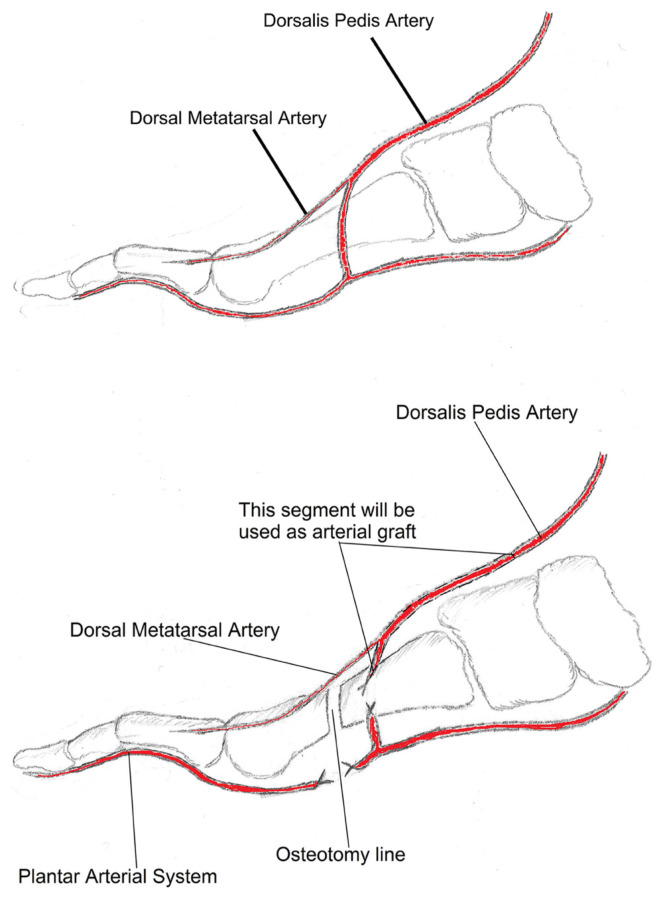
Schematic drawing of plantar dominant arterial system and the use of dorsalis pedis artery as an arterial graft.

The main technical difference is seen when the plantar dominant arterial system is present. Extensive plantar intermetatarsal dissection is not recommended in order not to increase the donor site morbidity. When the inter metatarsal artery is diminutive or absent, which means that plantar arterial system is dominant in this region, the dissection is continued by the plantar side about approximately 2 or 3 cm until metatarsophalangeal joint level and stop the plantar arterial dissection at this level. Instead of using vein graft as recommended, we prefer to use the dorsalis pedis artery as the arterial graft [5]. The dorsalis pedis artery is dissected distally until the main branch going down to the plantar arc between the first two metatarsals, and ligated just under the intermetatarsal muscle fascia. The diminutive metatarsal artery (if present) can be left intact. The dissection of the dorsalis pedis is continued proximally depending on the pedicle length required along with dorsal cutaneous veins and nerves included. Following the proximal and distal dissection of dorsalis pedis artery, the required tenotomies and osteotomies are performed, and the second toe flap is ready for transfer (Figure 1).

**Figure 2:**
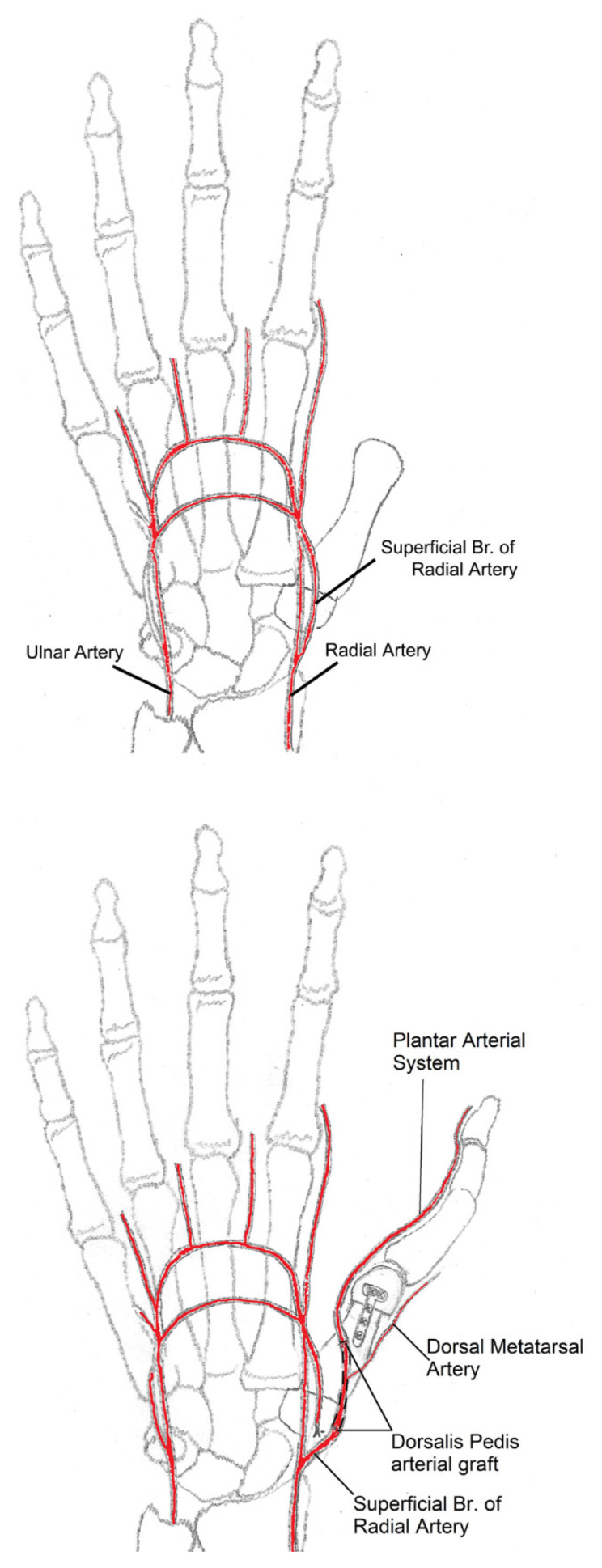
Schematic drawing of using superficial branch of radial artery as the receiving artery for anastomosis.

Depending on the level of osteotomy, the joint capsule, plantar plate and the ligaments of the transferred toe are connected to the corresponding structures at the recipient site of the hand. Osteosynthesis can be performed by various techniques such as K-wires, interosseous wiring, plates and screws, following determination of length, angle and rotation of the transferred toe. Flexor and extensor tendons are connected to the corresponding ones at the donor site with nonresorbable sutures. Next the microvascular anastomoses of the artery, vein and the coaptations of the nerves are performed. Dorsalis pedis artery is used as arterial graft during arterial anastomosis. Plantar mobilization of the dorsalis pedis artery is required at this step, which is very easily performed because of the loose fascial structures surrounding this vessel. The proximal end of the dorsalis pedis is anastomosed to the recipient donor artery. The distal end, which is the beginning of the intermetatarsal main branch going down to the plantar arch, is anastomosed to the previously dissected plantar digital arterial stump (Figure 2). Skin flaps are adjusted to the local skin, with great attention in order to prevent pressure increase the site of microvascular anastomoses, and skin grafts are applied when needed. Low molecular weight dextran (10 mg/kg i.v.) and acetylsalicylic acid (100 mg p.o.) were used for the first 5 days postoperatively, and then dextran was stopped and acetylsalicylic acid was continued for two more weeks.

## 3. Results

Eleven toe to hand transplantation cases in nine traumatic patients were included in this series. The mean age of the patients was 32.8 (between 6 and 57). All patients were male. One patient had diabetes mellitus and one patient had hypertension as a comorbidity. The reason for the operation was trauma in all patients. Second toe transfer was performed in all of the cases, followed by proximal ray resection of the donor toe. Two consecutive second toe transfers from each foot were performed in two cases with multiple finger amputations.

**Figure 3:**
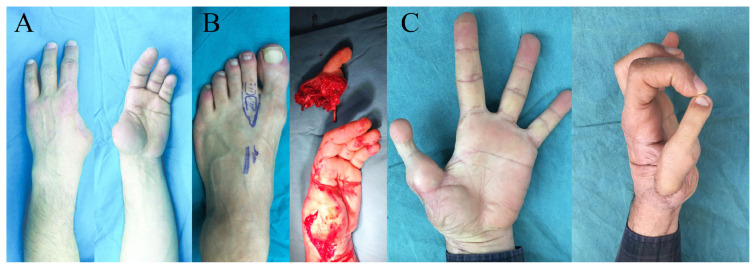
Views of patient 2. A 54-year-old male patient who has lost his first and second fingers of his left hand because of a severe hand trauma that happened 42 years ago. Thumb reconstruction was performed with the second toe of his left foot, which was transferred to the stump of the proximal phalanx of the thumb. The plantar arterial system was dominant in this patient and it was classified as type C according to Upton [5]. A: Preoperative view of the patient in case 2. B: The preparation of the second toe for transfer. C: Postoperative view of the patient in case 2.

**Figure 4:**
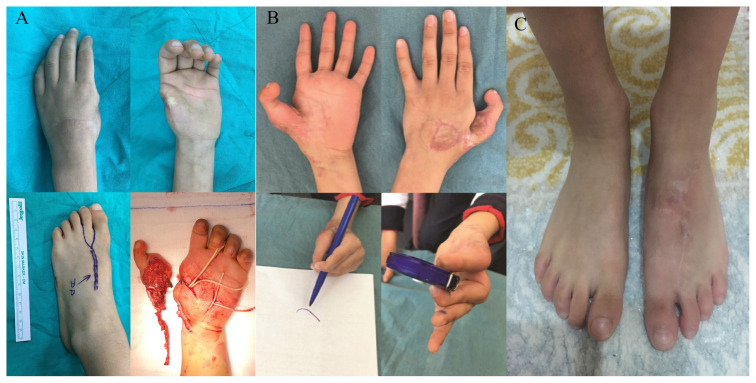
Views of patient 4. A 6-year-old boy who lost his left thumb in an accident when he was 2. Toe to thumb reconstruction was performed, and the second toe of his left foot was transferred to the stump of his left thumb. The arterial pattern of his second toe was found to be type D according to Upton in this patient [5]. A: Preoperative and intraoperative views of the patient in case 4. B: Postoperative views of the patient in case 4. C: Postoperative view of the donor foot of the patient in case 4.

Plantar dominant arterial system was seen in three of the nine patients (33.3%). Dorsal arterial system was dominant in the remaining patients. Dorsalis pedis arterial graft technique was used in these four cases. All of the toes survived, and reexploration was needed in two cases because of venous insufficiency. These two cases were totally salvaged after venous anastomosis revisions, which were performed within first 12 h postoperatively. Mean follow-up time was 16.4 months (between 12 and 24 months) (Table; Figures 3A–3C and 4A–4C).

## 4. Discussion

The thumb is the most functional finger on the hand. For this reason, thumb injuries cause serious loss of function. There are many studies on thumb reconstruction in the literature [6–10].

Toe-to-thumb transfer is considered the best among the thumb reconstruction options, but the dominant arterial system does not always have a stable anatomy in this technique [11,12]. The variations of the arterial patterns between the first two rays of the foot, seen during clinical toe dissections are classified into four groups according to Upton. While the dorsal system is dominant in types A and B (70%), the plantar system is dominant in types C and D (30%) [5].

Since the dorsal system is dominant in most of the patients [5], second toe transfers can mostly be done only depending on the dorsal system. Dissection of the second toe is initiated from the dorsal side of the first metatarsal web space, identifying the medial digital artery of the second toe. This artery arises from the first dorsal metatarsal artery and at this point. The caliber of this artery gives us an idea about the dominance of the dorsal or plantar arterial system. If the caliber of the medial digital artery of the second toe is good, the dissection is continued proximally to the first dorsal metatarsal artery and dorsalis pedis artery along with appropriate dorsal nerves and veins [5].

The patients, which are more rarely encountered, are the cases defined as types C and D in which the dorsal system is absent or very small [5], and the plantar system has to be preserved in these cases. The dissection of the plantar system is more challenging compared to the dissection of the dorsal system [4,13,14]. Plantar metatarsal artery dissection is performed from the plantar side of the foot, which can be facilitated by transaction of the transmetatarsal ligament. The proximal limit of the dissection can reach to the intermetatarsal cleft with effective retraction of the metatarsals. But on the other hand, this extensive dissection increases the morbidity of the donor foot and because of this, interpositional vein grafting is recommended instead of this extensive plantar dissection of the donor foot [5].

Saphenous vein or its branches are commonly used as vein grafts because of its accessibility. It is placed in a reversed position to allow free blood flow via the venous valves when used as vessel graft. Although vein grafts are often seen as a savior, the use of vein grafts also has disadvantages, requiring surgery in another healthy part of the body. On the other hand, one of the main problems with the use of saphenous vein grafting is the size discrepancy. The diameter of the saphenous vein is considerably larger than the medial and lateral digital proper arteries, and primary anastomosis of the great saphenous vein to the lateral proper digital artery is very difficult because of this size discrepancy. This can lead to thrombosis in the anastomosis. The muscular layer of veins is weaker than arteries and is more sensitive to hemodynamic instability. In cases where long vein graft use is required, hypotension may result in collapse and thrombosis in the vein due to weak bending resistance of vein graft [15–17].

There are plenty of studies in the literature comparing arterial grafts versus venous grafts. In the long term, arterial grafts show definite superiority for the graft patency compared to venous grafts [18,19]. Using dorsalis pedis artery as an vessel graft has many advantages such as; it does not necessitate a different surgical area such as leg to harvest saphenous vein, its diameter is more suitable than saphenous vein grafts that avoids size discrepancy [16] and as an arterial graft it is more resistant to pressure caused by edema and does not involve valves which can increase risk of thrombosis. In addition, the muscular layer in the dorsalis pedis artery is thicker and provides strong muscular tone. For this reason, it is more resistant to collapse in conditions such as hypotension and the probability of thrombosis is lower.

There are some limitations of this study. The sample size is relatively low. However, toe to hand transfers are seldom done operations and the mentioned plantar arterial dominancy is rarely seen. This technique has proved its versatility in these situations. Authors tried to express the complex operation with schematics. A missing comparison of preoperative imaging and intraoperative findings about dominant arterial system of the second toe is one of the limitations of this study. The preoperative visualization to evaluate anatomical variance of the second toe is the most useful way to avoid perfusion compromise of a toe flap. The surgeon will be able to make preoperative surgical plan precisely with these visualization methods. If the plantar arterial system is dominant the surgeon can easily adapt a dorsalis pedis arterial graft into surgical plan.

## 5. Conclusion

Plantar arterial system dominancy is a rarely seen situation that makes the difficult toe to hand transfer harder, and there is no alternative method other than vein grafting in the literature. This is the first study to recommend the use of dorsalis pedis as an arterial graft instead of vein grafts. In conclusion, we think that this surgical method described will make these rarely performed transfers easier and affect the success rates positively.

## Figures and Tables

**Table:** Characteristics of the patients included in the study.

Patient number	Age/Sex	Comorbidity	Primary cause	Recipient area	Dominant pattern	Complication	Follow-up (months)
**1**	36/Male	Diabetes mellitus	Trauma	Thumb	Dorsal	Venous insufficiency	18
**2**	54/Male	None	Trauma	Thumb	Plantar	None	18
**3**	33/Male	None	Trauma	Thumb – index finger	Dorsal	None	16
**4**	6/Male	None	Trauma	Thumb	Plantar	None	12
**5**	29/Male	None	Trauma	Thumb	Dorsal	None	14
**6**	57/Male	Hypertension	Trauma	Thumb	Dorsal	Venous insufficiency	17
**7**	27/Male	None	Trauma	Thumb – index finger	Plantar	None	24
**8**	26/Male	None	Trauma	Thumb	Dorsal	None	15
**9**	28/Male	None	Trauma	Thumb	Dorsal	None	14
